# Artificial intelligence and wheezing in children: where are we now?

**DOI:** 10.3389/fmed.2024.1460050

**Published:** 2024-08-27

**Authors:** Laura Venditto, Sonia Morano, Michele Piazza, Marco Zaffanello, Laura Tenero, Giorgio Piacentini, Giuliana Ferrante

**Affiliations:** ^1^Cystic Fibrosis Center of Verona, Azienda Ospedaliera Universitaria Integrata, Verona, Italy; ^2^Pediatric Division, Department of Surgery, Dentistry, Pediatrics and Gynaecology, University of Verona, Verona, Italy; ^3^Pediatric Division, University Hospital of Verona, Verona, Italy; ^4^Institute of Translational Pharmacology (IFT), National Research Council (CNR), Palermo, Italy

**Keywords:** wheezing, machine learning, artificial intelligence, asthma, digital health

## Abstract

Wheezing is a common condition in childhood, and its prevalence has increased in the last decade. Up to one-third of preschoolers develop recurrent wheezing, significantly impacting their quality of life and healthcare resources. Artificial Intelligence (AI) technologies have recently been applied in paediatric allergology and pulmonology, contributing to disease recognition, risk stratification, and decision support. Additionally, the COVID-19 pandemic has shaped healthcare systems, resulting in an increased workload and the necessity to reduce access to hospital facilities. In this view, AI and Machine Learning (ML) approaches can help address current issues in managing preschool wheezing, from its recognition with AI-augmented stethoscopes and monitoring with smartphone applications, aiming to improve parent-led/self-management and reducing economic and social costs. Moreover, in the last decade, ML algorithms have been applied in wheezing phenotyping, also contributing to identifying specific genes, and have been proven to even predict asthma in preschoolers. This minireview aims to update our knowledge on recent advancements of AI applications in childhood wheezing, summarizing and discussing the current evidence in recognition, diagnosis, phenotyping, and asthma prediction, with an overview of home monitoring and tele-management.

## Introduction

Wheezing is a musical sound, high-pitched and continuous, emitted from the chest during exhalation and resulting from the narrowing of the intrathoracic airway and expiratory flow limitation (1). The prevalence of wheezing disorders in preschool children varies worldwide and appears to have increased during the last decade (2). It is estimated that about one in three children experiences wheezing during the first 3 years of life (3). Viral infections trigger most wheezing episodes, involving up to 30–50% of preschool children (4). Generally, such episodes are mild and transient. However, one-third of preschoolers develop recurrent wheezing, which is defined as four or more episodes in the previous year (5). Recurrent wheezing has a significant impact on quality of life as well as on healthcare resources (6). Indeed, the economic burden of wheezing for the European Union is estimated at EUR 5.2 billion (7).

Artificial intelligence (AI) and machine learning (ML) encompass approaches such as data mining methodologies, predictive analytics, and advanced statistics for pattern recognition and neurocomputing (8). The application of AI technologies in paediatric allergology and pulmonology has increased, contributing to disease detection, risk profiling, and decision support (9, 10). Additionally, the COVID-19 pandemic has shaped healthcare systems, resulting in an increased workload and the necessity to reduce access to hospital facilities. Indeed, several studies have investigated the applications of AI and ML during the COVID-19 pandemic (11). Overall, such approaches could help address current issues in managing preschool wheezing, including phenotyping and improving parent-led/self-management, while reducing economic and social costs.

**Figure 1:**
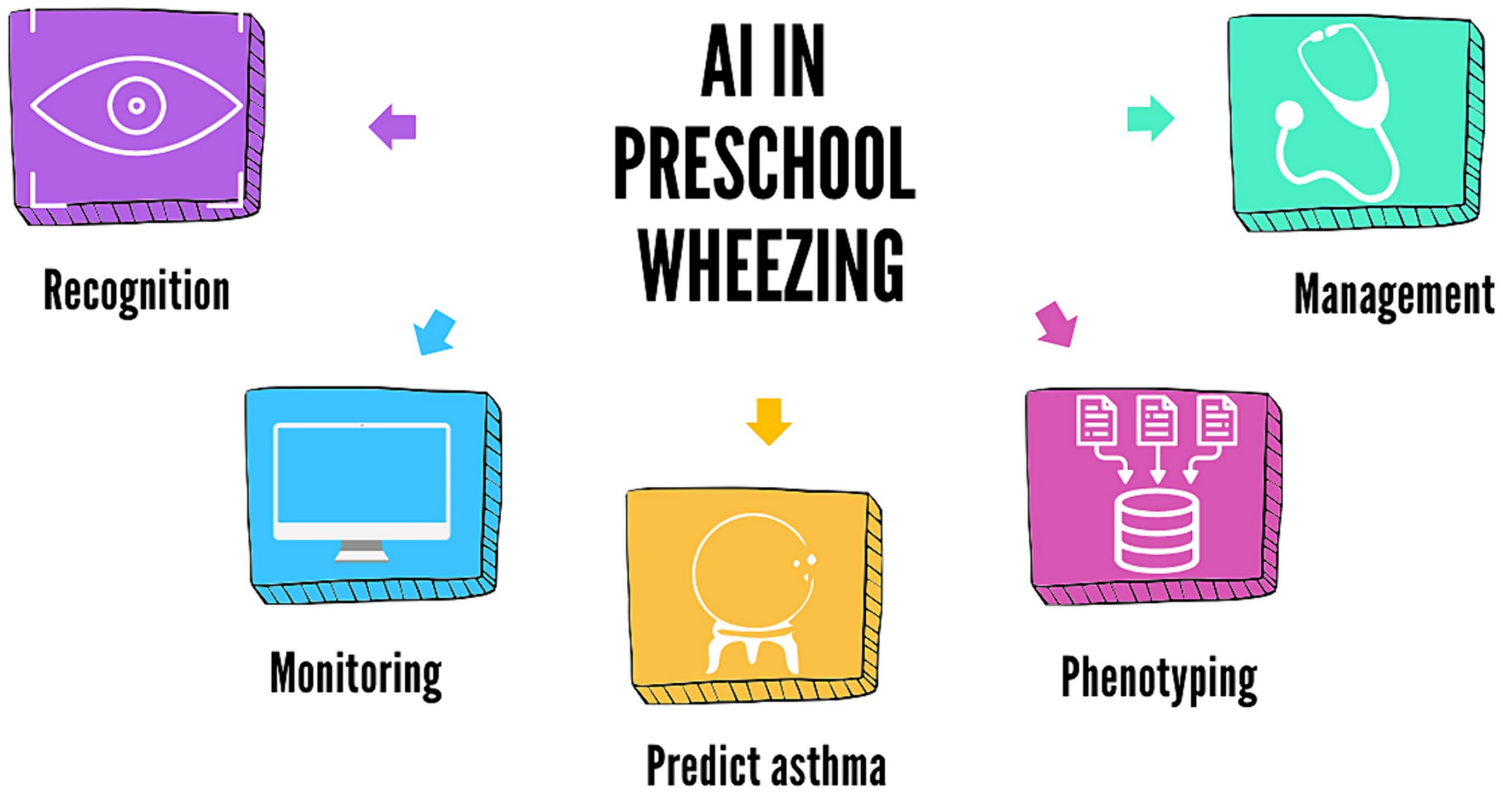
Possible applications of AI in preschool wheezing.

This minireview aims to summarize and discuss the current evidence on possible applications of AI in recognizing and monitoring wheezing in children and predicting future asthma development, progressing from deep phenotyping to patient-tailored management (Figure 1). We conducted a literature search in the PubMed database, selecting articles published over the last 10 years. We used medical subject headings (MeSH terms) and free-text terms related to wheezing, machine learning, artificial intelligence, and asthma and limited the search to clinical trials, randomized controlled trials, meta-analyses, and systematic reviews. Additionally, we manually consulted the reference lists of the retrieved articles. Manuscripts were selected by the authors (L.V. and S.M.), considering full manuscripts published in English in peer-reviewed journals.

## Can artificial intelligence recognize and monitor wheezing in children?

Parents and doctors often use “wheezing” to describe various respiratory sounds, such as crackles (12). The cheapest and non-invasive method for assessing wheezing is auscultation using a phonendoscope, which is operator-dependent and does not allow recording. Therefore, there is an increasing demand for an automatic, more objective, shareable, and reproducible method to assist doctors in diagnosing and monitoring patients with respiratory diseases (13).

The electronic stethoscope is an innovative version of the classic model, offering the ability to record and store chest sounds, allowing remote access. Many devices enable sound amplification, which is helpful for teaching (13). However, the digital data collected are subject to human interpretation and inter-operator variability, challenges that can be addressed by AI and ML technologies. These technologies have demonstrated good accuracy in recognizing respiratory sounds, particularly wheezing in children (Supplementary Table S1).

AI-assisted home stethoscopes can provide reliable information on asthma exacerbations. A recent study evaluated StethoMe for the automatic detection of pathological lung sounds (wheezes, crackles, and rhonchi) at home in 90 patients (0–18 years), demonstrating its efficacy in identifying asthma exacerbation across all ages (14). StethAid® is a device with a decision support system based on deep learning, an artificial neural network technology, used in an emergency department to recognize wheezing (15). The device recorded lung sounds from patients aged 2–18 years experiencing asthma exacerbation. These recordings were converted into spectrograms, serving as input for two deep learning models: ResNet-18 and Harmonic Networks. Both models were trained and validated to identify wheezing sounds from clear breathing sounds with good sensitivity, specificity, and accuracy. Specifically, ResNet-18 achieved 77% sensitivity, 70.1% specificity and 73.9% accuracy, while Harmonic Networks achieved 83.7% sensitivity, 84.4% specificity, and 84% accuracy.

A recent study by Ajay Kevat et al. (16) demonstrated high accuracy in recognizing children’s lung sounds using AI-enhanced digital stethoscopes, although differences were noted between various devices. AI stethoscopes can store diaries of wheezing episodes, enabling remote monitoring (14). Limitations of these devices include high cost, complexity of use, incompatibility with software and/or operating systems, and technical constraints (e.g., limited data memory, duration of autonomy, and varying frequency characteristics) (15).

Another device for analyzing lung sounds is PulmoTrack^®^ (17–19), which utilizes chest sensors with an external microphone to capture and cancel environmental noises. Its effectiveness was evaluated in a study (17) involving 120 infants during sleep, demonstrating that computerized wheezing detection is more objective, non-invasive, and standardized compared to medical auscultation. The device also proved beneficial in intensive care settings for managing patients with wheezing (20).

The HWZ-1000 T device (Omron Healthcare Corporation, Kyoto, Japan) was evaluated in a study (21) involving 374 children. Wheeze was detected by auscultation with a stethoscope and recorded using the wheeze recognition algorithm device (HWZ-1000T), based on the sound characteristics of wheezing. The device accurately identified wheezing (sensitivity 96.6%, specificity 98.5%, positive predictive value 98.3%, and negative predictive value 97.0%).

In the study by Dramburg et al. (22), 20 infants and preschool children (9–72 months) diagnosed with wheezing in the past year were recruited. All their families were requested to use the WheezeScan® digital wheeze detector (OMRON Healthcare Co., Ltd.) twice daily and simultaneously monitor the child’s respiratory symptoms through a smartphone clinical diary for 30 days. The results were displayed on an integrated screen that could be transmitted via Bluetooth to a PC or mobile device (e.g., smartphone or tablet). The study concluded that using the WheezeScan® Detector is straightforward and safe for children with wheezing. The support of a digital wheezing detector enhances parents’ self-efficacy in managing asthma and wheezing, boosting their confidence in handling their child’s wheezing at home. The WheezeScan^®^ demonstrated good sensitivity (83.3%) and specificity (100%) in wheezing recognition, albeit with limited visits (22). In a more recent study (23), the analysis of WheezeScan^®^ revealed no significant differences in wheeze control between study groups, with no impact on quality of life and minimal differences in parental efficacy in wheezing management.

Smartphone devices also play a role in integrating AI in Medical Practice. The ResAppDx^®^ algorithm (24) analyses cough using a microphone integrated into the smartphone, alongside symptoms reported by the patient and/or parents. Automated cough analysis has demonstrated good diagnostic accuracy for common childhood respiratory diseases and it is non-invasive and feasible even in resource-limited environments (24). Another smartphone-based algorithm for detecting cough sounds was evaluated (25), comparing training data that included recordings of children coughing and ambient audio with everyday noises. The algorithm achieved an accuracy of 99.7% and a specificity of 99.96% when tested on the coughs of 21 children between 0 and 16 years hospitalized for lung diseases. This suggests that smartphone applications can be used for clinical follow-up and as a digital endpoint in clinical trials (25).

AI algorithms for wheezing recognition have some limitations, such as difficulty in correlating certain respiratory sounds with specific illnesses and considering that paediatric cough sounds vary with age due to respiratory and vocal system development. It should also be acknowledged that digital devices remain limited when compared to traditional lung auscultation for patients with severe airflow obstruction, who may have silent lungs without wheezing (19), and that the effectiveness of a digital device can be influenced by various factors such as age and cultural background. In conclusion, while many studies highlight the effectiveness and applicability of AI digital devices in detecting wheezing, others have yet to achieve similar results. Therefore, more studies will be necessary to assess their effectiveness in recognizing wheezing in children.

## Can artificial intelligence predict asthma outcomes in children with wheezing?

Most children with asthma experience symptoms in early life, but these are typically transient, often disappearing by school age (6–13 years) (26). Therefore, it can be challenging to differentiate asthma from other wheezing disorders at this age (26). Over the last few decades, considerable effort has been dedicated to predicting asthma in children to identify earlier those at high risk and provide them with the best treatment option (26).

Previous studies based on population birth cohorts have identified distinct wheezing phenotypes (clusters) with associated early-life factors and outcomes, paving the path to predict wheezing trajectory and thereby develop targeted management (27). In this context, predictive models have been developed, considering various risk factors associated with asthma development, such as parental history of atopy and asthma, eczema and atopic dermatitis, allergic sensitization, and eosinophilia. However, they have rarely included environmental exposures and socioeconomic status (28). One of the most widely used models is the Asthma Predictive index (API) (29) and its modified version (mAPI) (30). Other models, such as the Isle of Wight score (31) and the Prevention and Incidence of Asthma and Mite Allergy (PIAMA) risk score, have also been developed (32); however, they have included children with recurrent chest infections, potentially misreporting episodes of wheezing (33).

Other predictive tools include the Leicester asthma prediction tool (34), the University of Connecticut (ucAPI) (35), and the Asthma Detection and Monitoring (ademAPI) (36), which also incorporate predictors such as 10 exhaled breath condensate biomarkers, 17 volatile organic compounds, and 31 genes. Although the ademAPI is the most comprehensive and sophisticated model, demonstrating reasonable specificity (88%) and sensitivity (90%), as well as the best positive and negative LRs (8.8 and 0.13, respectively), compared to other predictive models (33), its implementation in clinical practice remains challenging due to high cost (33). Even the original API, which includes only four items and a blood sample for eosinophil count, shows a good positive LR but a low negative LR, making it less effective in ruling out asthma (33). One of the more recent models is the CHILDhood Asthma Risk Tool (CHART) (37), which can identify children from 2 years of age at high risk of persistent wheezing and likely to develop asthma. Thanks to its simplicity, CHART could be used as a screening tool in primary care.

Overall, the models mentioned above indicate that the wheeze pattern alone cannot predict asthma progression. For this purpose, ML approaches have demonstrated better predictive performance and generalizability compared to regression-based models (27). In this context, artificial neural networks (ANNs) constitute a type of AI technique that learns the potential relationship between input–output mapping from a given dataset without prior knowledge or assumptions about the data distribution (38). This sets them apart from common statistical tests and makes them suitable for classification and prediction tasks.

One of the initial studies in this research field (26) employed Principal Component Analysis (PCA) for feature extraction, followed by the Least Square Support Vector Machine (LSSVM) classifier for pattern classification, resulting in a ML model with an accuracy of 95.54% in predicting asthma. More recently, a study (39) from the Isle of Wright birth cohort applied ML approaches to predict school-age asthma (at the age of 10 years) in early life (Childhood Asthma Prediction in Early life, CAPE model) and at preschool age (Childhood Asthma Prediction at Preschool age, CAPP model). Recursive Feature Elimination (RFE) with a random forest algorithm was used for feature selection. Seven ML classifiers were then implemented to identify the best classification algorithm: two Support Vector Machines (SVM), a decision tree, a random forest, Naive Bayes, Multilayer Perceptron, and K-Nearest Neighbours. Finally, the models were also validated in the Manchester Asthma and Allergy Study (MAAS) cohort. The SVM algorithms demonstrated the best performance for CAPE and CAPP, showing excellent sensitivity in predicting persistent wheezing. Interestingly, the study was implemented by incorporating genetic and epigenetic information (40), which marginally improved performance and indicated that genetic and epigenetic markers for the broader phenotype of “diagnosed with asthma” are unlikely to have clinical utility (41).

A limitation of using the scores mentioned above is the challenge of ruling out asthma rather than identifying it. However, in clinical practice, they can assist in identifying patients at high risk of developing asthma who are likely to respond to ICS, as shown in a latent class analysis (LCA) (42). This analysis showed that ICS treatment reduced exacerbations in children with persistent wheezing and conditions such as “sensitization with indoor pet exposure” and “multiple sensitization and eczema.”

Ultimately, the global diffusion of electronic health records (EHRs) created a need for automated chart review to diagnose asthma in children. Kaur et al. (43) developed a natural language processing (NLP) algorithm to identify children meeting API criteria. This NLP-API predicted asthma in preschoolers with a sensitivity of 86%, specificity of 98%, positive predictive value of 88%, and negative predictive value of 98%. Such an index has the potential to be utilized by healthcare systems to identify children meeting API criteria, even in early childhood (e.g., < 3 years old), thereby improving access to preventive and therapeutic interventions for asthma and monitoring their outcomes (9, 43).

Nonetheless, using AI algorithms and ML for predicting asthma outcomes in children may raise potential ethical concerns. Firstly, AI algorithms are trained on a large volume of personal data from EHRs, including clinical, imaging, and even genomic data, so it appears clear that ensuring privacy is critical, while overprotection of the data collection, usage, and sharing can slow down the innovation in AI training (44). To overcome this important limitation and preserve privacy, new techniques are emerging in AI such as the generation of synthetic data that mirrors the real-world dataset, but even this approach can not ensure full privacy, especially in small datasets, as patients from a specific region (45) or in a particular age range. Moreover, if AI algorithms are trained in a limited dataset, they can inadvertently present some gender, socioeconomic, and ethnic bias, that can exacerbate health inequalities in underrepresented social groups (44, 46), resulting in incorrect predictions, and leading to misdiagnosis when these biases are not corrected or prevented during the elaboration of the training dataset (44).

For these reasons, taking also in consideration that AI can actually make mistakes, AI can not be held morally accountable, having a role only as a decision support aid for clinicians (44). If used in clinical practice to provide therapeutic recommendations, to inform prognosis or risk of future events, informed consent should be provided to patients, explaining to them if AI has been used, clarifying which type of AI and how it was involved in the decision process, informing also about potential pitfalls (47).

## Can artificial intelligence identify wheezing endotypes in preschool children?

Wheezing has been classified into different phenotypes since the first population-based cohort studies aimed to understand its heterogeneity (41).

The initial study was the Tucson Children’s Respiratory Study (5), which identified three patterns of preschool wheezing (early transient, late-onset, and persistent), each associated with different risk factors. Subsequent studies have further defined additional phenotypes and temporal patterns, such as the Avon Longitudinal Study of Parents and Children (ALSPAC) (4, 48), the Prevention and Incidence of Asthma and Mite Allergy (PIAMA) birth cohort (49), the Viva project (50).

This approach assumes that patterns of symptoms and/or biomarkers assessed in longitudinal or cross-sectional studies reflect the underlying mechanisms, leading to the identification of asthma endotypes, but this assumption is uncertain (51).

ML approaches such as LCA have also been used in preschool wheezing (4, 52–54) and childhood asthma (55, 56).

An interesting study (57) focused on the longitudinal trajectory of wheezing exacerbations using an ML approach (k-means clustering), which identified two types of trajectories from birth to adolescence. The k-means clustering revealed that a shorter duration of breastfeeding was one of the early risk factors for frequent exacerbations. Additionally, children with frequent exacerbations showed increased airway resistance and, at 8 years of age, a lower lung function with higher FeNO levels, with evolution to asthma at 16 years of age.

ML approaches have also contributed to identifying specific genes, as demonstrated by Lin et al. (58), who employed Weighted Gene Co-expression Network Analysis (WGCNA) to identify gene co-expression modules associated with pediatric asthma. They subsequently used ML algorithms (random forest, lasso regression algorithm, and support vector machine with recursive feature elimination) to classify asthma cases and controls based on the 11 identified genes that can potentially explain the pathophysiology of difficult asthma and serve as biomarkers for diagnosis and targets for future advanced treatments. 

Notably, as Saglani et al. (51) highlighted, we should be cautious about assuming that clusters identified in these studies represent “true” wheezing endotypes. The limitations of these studies include the identification of different risk factors for the same disease (wheezing) using the same technique (LCA), differences in the characteristics of the wheezing trajectories, the temporal description of wheezing in these clusters that may not align with the temporal presentation of symptoms, and ultimately, the diverse pathological mechanisms that can lead to wheezing within the same cluster. For example, persistent wheezing can arise from recurrent airway infections due to impaired immunological responses or from allergen sensitization and exposure (51).

Considering these limitations, ML has identified more intermediate phenotypes with one certainty across all studies: all wheezing phenotypes, even the transient ones, lead to impaired lung function in early adulthood (41). Moreover, the results obtained so far need validation in further longitudinal studies involving larger populations of preschool children.

## Conclusion

The applications of AI in preschool wheezing have encompassed various research topics, including phenotyping, delineating trajectories using data from EHR, predicting future asthma development and exacerbations, and identifying early risk factors and genetic markers. There are also several applications for clinical practice, such as wheezing recognition using AI-augmented stethoscopes or smartphones and telemonitoring (59).

Although AI could support clinicians in their daily practice, some questions must be addressed, especially when caring for children. Regulatory requirements are of foremost importance in protecting sensitive data and maintaining privacy. Additionally, AI approaches and their results must be rigorously validated before we adopt them in our routines.

In conclusion, AI could enhance the management of preschool wheezing, from recognition to identifying children potentially at high risk of exacerbation and asthma, thereby enabling an AI-tailored treatment. Furthermore, we should consider AI’s utility in case of future pandemics, particularly in telemonitoring and telemanagement. However, we must be mindful of its limitations and work to address them to ensure the safety of children’s data.

## Author contributions

LV: Conceptualization, Methodology, Writing – original draft, Writing – review & editing. SM: Methodology, Writing – original draft, Writing – review & editing. MP: Writing – original draft, Writing – review & editing. MZ: Writing – original draft, Writing – review & editing. LT: Writing – original draft, Writing – review & editing. GP: Writing – original draft, Writing – review & editing. GF: Conceptualization, Methodology, Writing – original draft, Writing – review & editing.
