# Supplementary material for: Artificial intelligence and wheezing in children: where are we now?
Source: Front Med (Lausanne). 2024 Aug 27;11:1460050. doi: 10.3389/fmed.2024.1460050 (PMC11385867; doi:10.3389/fmed.2024.1460050)
Supplement: Supplementary file 1 [file Table_1.docx]

Supplementary Material

Artificial intelligence and wheezing in children: where are we at?

**Laura Venditto^1,2^, Sonia Morano^2^, Michele Piazza^2^, Marco Zaffanello^2^, Laura Tenero^3^, Giorgio Piacentini^2^, Giuliana Ferrante^2*^**

^1^Cystic Fibrosis Center of Verona, Azienda Ospedaliera Universitaria Integrata, Verona, Italy

^2^ Department of Surgery, Dentistry, Pediatrics and Gynaecology, Pediatric Division, University of Verona, Verona, Italy

^3^Pediatric Division, University Hospital of Verona, Verona, Italy.

*** Correspondence:**Corresponding Author
michele.piazza@univr.it

# Supplementary Table 1 Studies on digital auscultation published in the last decade

| **Study (year)** | **Country** | **Type of study** | **Aim of the study** | **Methods** | **Study population (male%)** | **Study population’s age** | **Main findings** | **Parents satisfaction** | **Quality of life** | **Performance**  **Sensitivity specificity accuracy** |
| --- | --- | --- | --- | --- | --- | --- | --- | --- | --- | --- |
| Do et al. (2024)(1) | Germany, Turkey and United Kingdom | Multicentre randomised controlled open-label trial | Assess if a digital support tool for wheeze recognition improves symptom control | Digital wheeze detector WheezeScan™ with a mobile application WheezeMonitor™ | 167 children; 87 intervention group (69.5%) | Mean 3.2 years (SD 1.6) | No statistically significant difference in wheeze control assessed by TRACK | PACQLQ and PAMSES improved without statistical difference between the groups | TAPQOL improved without statistical difference between the groups | Sensitivity 100%*  Specificity 95.7%* |
| Dramburg et al. (2021)(2) | Germany | Single-armed pilot study | Assess the feasibility of a digital support tool for wheezing recognition | Digital wheeze detector WheezeScan™ with a mobile application WheezeMonitor™ | 20 (85%) | Mean 39.5 months (q1:  24.3 months; q3: 60 months) | Improvement of ACT in 64% | Improvement of PAMSES | NI | PPV of 83.3% and NPV of 87.3% compared to the doctors’s judgement. |
| Habukawa et al. (2020)(3) | Japan | Case control study | Develop an algorithm for automatic wheeze recognition | Digital wheeze detector (future WheezeScan™) | 214 (64.5%) | Mean 57.5 months (SD 43.1 months) | Development of an accurate algorithm for wheezing recognition | NI | NI | Sensitivity 100%, specificity 95.7%,  PPV 90.3%,  NPV 100%. |
| Kim et al. (2022) (4) | Korea | Prospective study | Develop an improved deep-learning model learning to detect wheezing in children | Electronic stethoscope (Jabes, GSTechnology, Seoul, Korea) | 76 (**) | (**) | 34-layers ResNet with CBAM + Tabular  Data enhibited the highest performance when compared to other models | NI | NI | Accuracy 91.2%,  AUC 89.1%,  precision 94.4%,  recall of 81%,  F1-score of 87.2%. |
| Porter et al. (2019)(5) | Australia and New Zealand | Prospective, multi-centre study | Compare diagnoses made by the algorithm to those from  a clinical adjudication panel | Automatic  cough detector  using Time Delay Neural (TDNN) Network  operating and identifying Mel Frequency Cepstral  Coefficients (MFCC) | 585 (59%) | Mean 53 months (SD 37 months) | The analyser  was able to diagnose reactive airway disease at a high-performance  level without the need for bronchodilator-response testing | NI | NI | Focusing on asthma and reactive airways diseases, the  algorithm achieved excellent agreement results  (PPA97%, NPA 91%) |
| Kruizinga et al. (2022)(6) | The Netherlands | Prospective validation study | Develop a smartphone-based algorithm that objectively and automatically counts cough sounds of children | CHDR MORE® application on a smartphone | 21 children (**) | 0–16 years (**) | Good accuracy on detection of cough sounds | NI | NI | Accuracy 99.7%, Sensitivity 47.6%  Specificity 99.96%  PPV 82.2%  NPV 99.8% |
| Liao et al (2022)(7) | China | Validation study | Classify bronchitis and pneumonia in children by  analyzing cough sounds | Classification Framework based on Cough Sounds (CFCS) adopting Support Vector Machine (SVM) and t Long Short-Term Memory Network (LSTM) | 173 children  (54%) | 0-11 years (**) | CFCS can  effectively classify children into bronchitis and pneumonia in children | NI | NI | SVM: accuracy of 86.04%  Precision and recall of bronchitis: 93.75%, 88.24%  Precision and recall of pneumonia: 87.5%, 93.33%.  AUC of SVM: 0.92  AUC of LSTM: 0.93 |
| Kevat et al. (2020)(8) | Australia | Prospective study | Test an AI algorithm to detect crackles and wheeze in children | Recordings collected using two digital stethoscopes (Clinicloud™ and Littman™) submitted for analysis by a blinded AI algorithm (stethome AI) | 25 (72%) | Median age 6.7 years (interquartile range 3.4) | AI can detect wheeze with good accuracy | NI | NI | Focusing on wheeze detection:  PPA 0.90 (Clinicloud), and 0.80 (Littman)  NPA 0.97 (Clinicloud),and 0.95 (Littman) |
| Arjoune et al. (2023) (9) | USA | Multicenter Prospective Study | Validation of StethAid (a digital platform for AI-assisted auscultation and telehealth) | StethAid, consisting of a wireless digital stethoscope, mobile applications, customized patient-provider portals, and deep learning algorithms | NS (**) | 2-18 years (**) | Accurate detection of wheezing | NI | NI | Focusing on wheeze detection:  Harmonic Networks  sensitivity 83.7%  specificity of 84.4%  accuracy of 84.0%.  ResNet18:  sensitivity 77.0%, specificity of 70.1%, accuracy of 73.9% |
| Puder et al (2016)(10) | Germany | Retrospective study | Evaluate the quality of respiratory sound recordings in young infants and determine whether the position of the sensor affected computerized wheeze detection. | PulmoTrack® Model 2020 | 112 (57%) | Median age 144 days (100–203) | TR sensor was less often affected by disturbances than the CW sensor, with a better quality of respiratory sound recordings. | NI | NI | Sounds from the chest wall were more often  affected by disturbances than sounds from the trachea (23% versus 6%, p < 0.001). |
| Puder et al (2014) (11) | Germany/Israel | Prospective study | To determine and validate optimal cut-off values for computerized wheeze  detection, based on the assessment by trained clinicians of stored records of lung sounds, in infants aged <1 year | PulmoTrack® Model 2020 | 120 (70%) | Median age 153 days (107–273) | PulmoTrack® can  detect wheezing in neonates, with good values of sensitivity and specificity for  inspiratory and expiratory wheezes.  Computerized wheeze detection reliably detects  even short periods of wheezing. | NI | NI | Sensitivity 85.7% for inspiratory wheezes and 84.6% for expiratory wheezes.  Specificity of 80.7% for inspiratory wheezes and 82.5% (expiratory wheezes. |
| Zhang et al (2021)  (12) | China | Prospective study | Evaluate the use of AI algorithm for detecting breath sounds in children with pulmonary diseases. | Class II CE-marked electronic  stethoscope (Yunting model II, Tuoxiao, Shanghai, China) | 112 (73.2%) | Median age 12.5 months (q 25% 5.0, q 75% 41.8) | The precision, the specificity and the F1 score in the detection of wheezing of the AI were significantly higher when compared to general pediatrician, with the highest accuracy in younger than 12 months | NI | NI | Sensitivity 86.4%  Specificity 83.0%  Precision 76%  F1 score 80.9% |
| Yu et al (2020) (13) | China | Retrospective | Compare machine learning–based models developed to identify asthma | CatBoost, Logistic Regression, Naïve Bayes, and Support Vector Machines (SVM) | DataSet-1   3,761 cases, DataSet-2   2,123 cases  (**) | 0-14 years  DataSet-1 positive group mean ± SD, 3.680 ±2.6891    DataSet-1 negative group mean ± SD 2.176±2.8111  DataSet-2 positive group mean ± SD 3.605±2.7956.  DataSet-2 negative group mean ± SD 4.975±4.0442 | CatBoost model outperformed other models. AI model could rapidly and accurately identify asthma in general medical wards of children, and may aid primary pediatricians in the correct diagnosis of asthma | NI | NI | Accuracy of 84.7% and an area under the curve (AUC) of 90.9% on TestSet-1  Accuracy of 96.7% and an AUC of 98.1% on TestSet-2 |
| Habukawa et al (2023) (14) | Japan | Prospective study | To validate the developed automatic wheeze recognition algorithm as a clinical medical device in children at different institutions | HWZ-1000T, Omron Healthcare Corporation, Kyoto, Japan | 374 (64.4%) | Mean 44.3 months, (SD 31.6) | The wheeze recognition algorithm was verified to identify wheezing with high accuracy; and, it might be useful in the practical implementation of asthma management at home. It will be useful for wheezing management at home and in remote medical care. | NI | NI | Sensitivity 96.6% Specificity 98.5% Positive Predictive Value 98.3%  Negative Predictive Value 97.0% of the wheeze recognition algorithm |
| [Emeryk](https://pubmed.ncbi.nlm.nih.gov/?term=Emeryk%20A%5BAuthor%5D) et al (2023)(15) | Poland | Observational study | Determine which devices and parameters work better in detecting asthma exacerbations | Home stethoscope, peak flow meter, pulse oximeter and subjective breathing quality | 90 children and  59 adults | 52 children aged 0-5 years: mean 3.0 years (q1 3.0, q3 4.25)  38 children aged 6-17 years:  mean 8.5 years (q1 7.0, q3 10.0)  59 adults, mean 38.0 years  (q1 32.5, q3 43.0) | A set of parameters measured by an AI-aided home stethoscope allows for the detection of asthma exacerbations without the need for performing PEF measurements. | NI | NI | AUC 84% [95% CI, 82%-85%] |

** Parameters derived from a previous study (Habukawa C, Ohgami N, Matsumoto N, Hashino K, Asai K, Sato T, Murakami K. A wheeze recognition algorithm for practical implementation in children. PLoS One. 2020 Oct 8;15(10):e0240048. doi: 10.1371/journal.pone.0240048. PMID: 33031408; PMCID: PMC7544038.)*

*** Sex and/or age are not clearly explicated in the text*

***TRACK****: Test for Respiratory and Asthma Control in Kids;* ***TAPQOL****: disease-specific quality of life;* ***PACQLQ****: parental quality of life;* ***PAMSES****: parental asthma management self-efficacy;* ***PPV****: positive predictive values;* ***NPV****: negative predictive values;* ***ACT****: asthma control test;* ***NI****: not investigated;* ***SD****: standard deviation;* ***PPA*** *Positive Percent Agreement;* ***NPA*** *Negative Percent Agreement;* ***AI*** *artificial intelligence;* ***NS*** *not specified;* ***F1 score***

**References**

1. Do YH, van Aalderen W, Dellbrügger E, Grenzbach C, Grigg J, Grittner U, Haarman E, Hernandez Toro CJ, Karadag B, Roßberg S, et al. Clinical efficacy and satisfaction of a digital wheeze detector in a multicentre randomised controlled trial: the WheezeScan study. *ERJ Open Res* (2024) 10:00518–02023. doi: 10.1183/23120541.00518-2023

2. Dramburg S, Dellbrügger E, van Aalderen W, Matricardi PM. The impact of a digital wheeze detector on parental disease management of pre-school children suffering from wheezing-a pilot study. *Pilot Feasibility Stud* (2021) 7:185. doi: 10.1186/s40814-021-00917-w

3. Habukawa C, Ohgami N, Matsumoto N, Hashino K, Asai K, Sato T, Murakami K. A wheeze recognition algorithm for practical implementation in children. *PLoS One* (2020) 15:e0240048. doi: 10.1371/journal.pone.0240048

4. Kim BJ, Kim BS, Mun JH, Lim C, Kim K. An accurate deep learning model for wheezing in children using real world data. *Sci Rep* (2022) 12:22465. doi: 10.1038/s41598-022-25953-1

5. Porter P, Abeyratne U, Swarnkar V, Tan J, Ng T, Brisbane JM, Speldewinde D, Choveaux J, Sharan R, Kosasih K, et al. A prospective multicentre study testing the diagnostic accuracy of an automated cough sound centred analytic system for the identification of common respiratory disorders in children. *Respir Res* (2019) 20:81. doi: 10.1186/s12931-019-1046-6

6. Kruizinga MD, Zhuparris A, Dessing E, Krol FJ, Sprij AJ, Doll R-J, Stuurman FE, Exadaktylos V, Driessen GJA, Cohen AF. Development and technical validation of a smartphone-based pediatric cough detection algorithm. *Pediatr Pulmonol* (2022) 57:761–767. doi: 10.1002/ppul.25801

7. Liao S, Song C, Wang X, Wang Y. A classification framework for identifying bronchitis and pneumonia in children based on a small-scale cough sounds dataset. *PLoS ONE* (2022) 17:e0275479. doi: 10.1371/journal.pone.0275479

8. Kevat A, Kalirajah A, Roseby R. Artificial intelligence accuracy in detecting pathological breath sounds in children using digital stethoscopes. *Respir Res* (2020) 21:253. doi: 10.1186/s12931-020-01523-9

9. Arjoune Y, Nguyen TN, Salvador T, Telluri A, Schroeder JC, Geggel RL, May JW, Pillai DK, Teach SJ, Patel SJ, et al. StethAid: A Digital Auscultation Platform for Pediatrics. *Sensors (Basel)* (2023) 23:5750. doi: 10.3390/s23125750

10. Puder LC, Wilitzki S, Bührer C, Fischer HS, Schmalisch G. Computerized wheeze detection in young infants: comparison of signals from tracheal and chest wall sensors. *Physiol Meas* (2016) 37:2170–2180. doi: 10.1088/0967-3334/37/12/2170

11. Puder LC, Fischer HS, Wilitzki S, Usemann J, Godfrey S, Schmalisch G. Validation of computerized wheeze detection in young infants during the first months of life. *BMC Pediatr* (2014) 14:257. doi: 10.1186/1471-2431-14-257

12. Zhang J, Wang H-S, Zhou H-Y, Dong B, Zhang L, Zhang F, Liu S-J, Wu Y-F, Yuan S-H, Tang M-Y, et al. Real-World Verification of Artificial Intelligence Algorithm-Assisted Auscultation of Breath Sounds in Children. *Front Pediatr* (2021) 9:627337. doi: 10.3389/fped.2021.627337

13. Yu G, Li Z, Li S, Liu J, Sun M, Liu X, Sun F, Zheng J, Li Y, Yu Y, et al. The role of artificial intelligence in identifying asthma in pediatric inpatient setting. *Ann Transl Med* (2020) 8:1367. doi: 10.21037/atm-20-2501a

14. Habukawa C, Ohgami N, Arai T, Makata H, Tomikawa M, Fujino T, Manabe T, Ogihara Y, Ohtani K, Shirao K, et al. Wheeze Recognition Algorithm for Remote Medical Care Device in Children: Validation Study. *JMIR Pediatr Parent* (2021) 4:e28865. doi: 10.2196/28865

15. Emeryk A, Derom E, Janeczek K, Kuźnar-Kamińska B, Zelent A, Łukaszyk M, Grzywalski T, Pastusiak A, Biniakowski A, Szarzyński K, et al. Home Monitoring of Asthma Exacerbations in Children and Adults With Use of an AI-Aided Stethoscope. *Ann Fam Med* (2023) 21:517–525. doi: 10.1370/afm.3039

**
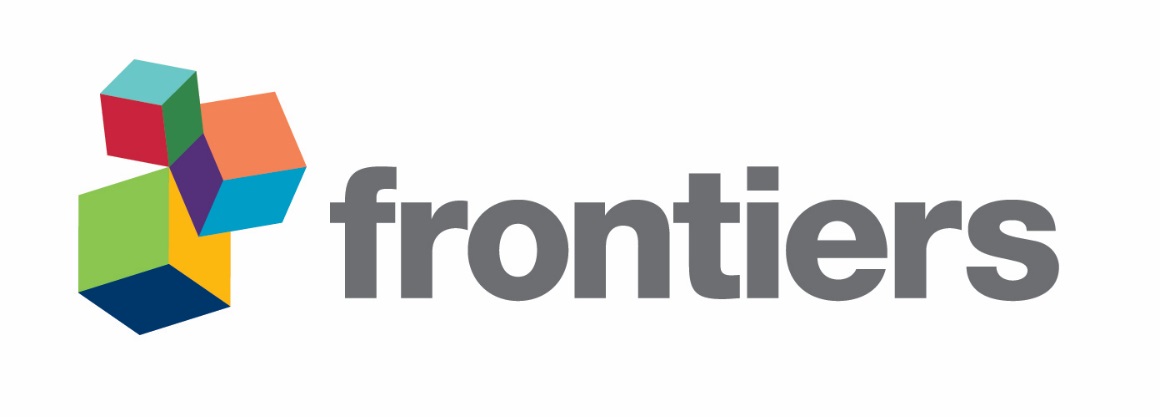
**
